# Complications of diabetes in China: health system and economic implications

**DOI:** 10.1186/s12889-019-6569-8

**Published:** 2019-03-06

**Authors:** Wenhui Mao, Chi-Man Winnie Yip, Wen Chen

**Affiliations:** 1 School of Public Health, Fudan University, 138 Yi Xue Yuan Road, P.O. Box 187, Shanghai, 200032 China; 2 Duke Global Health Institute, Duke University, Rm 209, 310 Trent Drive, Durham, NC 27710 USA; 3 Department of Global Health and Population, Harvard T.H Chan School of Public Health, Harvard University, 665 Huntington Avenue, Building 1 Room 1210C, Boston, MA 02115 USA

**Keywords:** Diabetes and its complications, Economic burden, Prevention, Health system

## Abstract

**Background:**

The prevalence of diabetes and diabetic complications increased alarmingly which also brought heavy burden to patients and health system.

**Methods:**

We used mix approaches to summarize evidence from published articles and policy documents on the extent and trends of diabetic complications, potential causes, and awareness and services utilization of diabetes in China.

**Results:**

The annual direct medical expense per patient varied among different types of complications and increased dramatically with the number of diabetic complication and patients were exposed to great financial risk. The number of health policies and strategies on diabetes and its complications at the national level is limited. Primary and secondary preventions such as health education and early diagnosis are necessary.

**Conclusions:**

With an increasingly burden of non-communicable diseases such as diabetes and its complications, efforts should be invested in education, early screening mechanism and patient management programs to improve the primary and secondary prevention of diabetes and its complications. An integrated services delivery system centered on primary level is recommended to promote education, early case-detection and screening, patient management, referral and care-coordination between primary, secondary and tertiary health care providers.

**Electronic supplementary material:**

The online version of this article (10.1186/s12889-019-6569-8) contains supplementary material, which is available to authorized users.

## Background

In 2014, one person died from diabetes in every 7 s, leading to 4.9 million of deaths worldwide [1]. Diabetes also added the risk of complications and premature death in the general population [2, 3]. In the United States, half of the patients with diabetes died of cardiovascular disease (a complication of diabetes) [4]; diabetic retinopathy (DR), another common complication of diabetes, was the leading cause of blindness among working-age population [5]. According to the American Diabetes Association, USD 27 billion was spent in 2007 in the US for treating diabetes and another USD 58 billion for diabetes-related chronic complications [6].

China witnessed a marked increase in the prevalence rate of diabetes, from 2.5% in 1994 [7] to 9.7% in 2008 [8] and further to 11.6% in 2010 [9], amounting to an increase of 3000 new patients per day [10]. Moreover, 15.5% of the adult Chinese population had pre-diabetes [8]. Among patients with diabetes, 76.4% reported at least one kind of complication [11] which has proven to be the leading cause of death in those with diabetes [12]. The increased patients with diabetes have also contributed to the growth of health expenditure in China. Health expenditure on diabetes was USD 0.25 billion in 1993 (or 0.07% of GDP) and it had dramatically increased to USD 8.65 billion in 2008 (0.21% of GDP) [11]. The average annual growth rate of health expenditure for type 2 diabetes was 19.9% between 1993 and 2003 in China, compared to 12.8% of growth rate of GDP or 12.9% of growth rate of the total health expenditure [13, 14]. It is also reported that health expenditure of patients with complications was 3.36 times higher than those without complications in China [15, 16].

Urbanization, diet and decreasing levels of physical activity, with a consequent epidemic of obesity have contributed to the rapid increase of diabetes in China [17, 18]. Yet significant gaps exist in people’s knowledge about diabetes and its prevention. 60.7% of adults with diabetes had not previously been diagnosed [8] and 66.7% of diabetes patients had not received regular examination as recommended by clinical guideline [19]. A comparison of out-of-pocket payment for diabetic treatment relative to annual average income in different regions of China showed that patients with diabetes in rural or less developed regions suffered from higher out-of-pocket payment [11, 20, 21].

Despite the rapid increases in the prevalence and economic burden of diabetes and its complications as well as the existence of cost-effective interventions [22], national strategy or special financing mechanism to meet these challenges is absent. It was identified that patients with lower socioeconomic status (SES) experience worse clinical outcomes than those with better SES which lead to equity issue of the health system [23]. This review aims to describe the trends of diabetes (DB) and its complications (DBC), their challenges to the health system and economic implications in China. We first review evidence on the extent and trends of DB/DBC and potential causes. We next present evidence on the costs associated with DB/DBC, followed by an analysis of how existing health systems address DB/DBC. In the last section, we conclude and draw policy recommendations on the development of public health, service delivery and financing system of China to prevent and treat DB/DBC.

## Methods

We used mix approaches to summarize evidence on the extent and trends of diabetic complications, potential causes, and awareness and services utilization of diabetes in China. We performed a review on published articles and policy documents with case-study as supplement. In addition, we conducted key informants interviews to obtain the opinions and recommendations on how to address the challenges and issues in diabetes control and care.

To collect evidence about the epidemiology characters, service use and economic burden of DB/DBC, we performed a systematic review. We first conducted a pilot search in PubMed and then we finalized the searching strategy and conduced searching in PubMed, Cochrane library, EMBASE (OVID), Elsevier Science Direct, EBSCO, Web of Science, Wiley database for articles in English and the Wan Fang and China Academic Journals (CNKI) database for articles in Chinese published before December 2017. We collected policy documentations about the management of diabetes through local contacts. A list of full searching strategies for all databases can be found at Additional file 1.

Inclusion criteria were: 1) Types of publications: peer-reviewed research, PhD dissertations, policy documents and reports; 2) Language: English or Chinese; 3) Study design: clear description on study design should be provided, and we include cross-sectional study, case-control study, cohort study and pilot study; 4) Data collection method: data collection method should be described clearly and we include population-based survey, facility survey, patient medical records or biomedical tests; 5) Study population: general population, or Type I or II diabetes patients diagnosed by certificated personnel (doctors/physicians).

We excluded commentary, editorial, letter to the editor, systematic review, narrative review and meta-analysis review; studies without clearly described methods or had less than 50 patients were also excluded.

Two reviewers (WM and WC) first screened the titles and abstracts of the articles and identified the eligibility of literatures based on the inclusion and exclusion criteria. We then extracted information about study design, epidemiology characters, service utilization and economic burden for further review. Policy recommendations were formed based on the key findings of literatures.

**Fig. 1:**
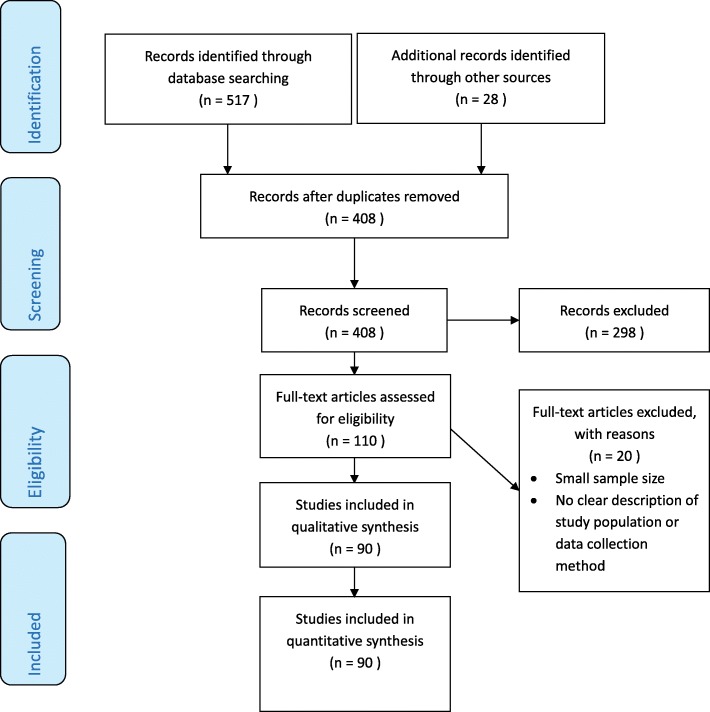
PRISMA flow chart

Figure 1 (PRISMA flow-chart) presented an illustration of the searching output. The initial search yielded 517 potentially relevant articles and narrowed down to 110 studies after screening on abstracts and titles. Ninety articles finally met all inclusion criteria and were reviewed by full text. Among all reviewed articles, about half were published between 2006 and 2012

## Results

### Trends in DBC

#### Prevalence of diabetic complications

The overall prevalence of diabetic complications were consistently reported at a high level in China (Table 1). During 2007–2008, a survey on outpatient visits indicated that 52.0% of diabetes patients had at least one complication [24]. For patients hospitalized for diabetes, 86.3–90.7% had at least one, 80.0% had at least two and 46.7% had at least three complications [25–27]. With regard to the prevalence of chronic diabetic complications across systems, 30.5% had single-system complications while 15.4, 4.9 and 1.3% had complications across 2, 3 and over 4 systems respectively [24].

**Table 1:** Prevalence of Diabetic Complications in China from Published Research

Observation Period	Study Site	Study Population	Prevalence of Complications	
			Overall	Different Kind of Complications
1980s [31]	2 northern cities	447 patients with diabetes		Proteinuria: 57.1%; Retinopathy: 47.4%
1986 [29]	1 northern city (Da Qing Study)	423 newly diagnosed patients with diabetes		Microaneurysms and/or small intraretinal haemorrhage: 15.4%; Soft exudates: 5.5%; Hard exudates:7.1%; Proliferative retinopathy: 2.3%
2001 [43]	1 northern city (Beijing Eye Study)	4439 inhabitants over 40 years old		Retinopathy: 6.5%
1998 and 2006 [24]	Throughout China (Diabcare Study)	2246 diabetes patients in 1998 and 2702 in 2006		Neuropathy & retinopathy: 84% in 1998 and 78% in 2006
2007 [25, 26]	20 hospitals from 4 cities	1524 T2D outpatients	52.0% ≥1	Macrovascular: 33.4%; Microvascular: 34.7%; Cardiovascular: 30.1%; Nephropathy:10.7%; Foot disease: 0.8%; Ocular lesions: 14.8%; Cerebrovascular conditions:6.8%; Neuropathy:17.8%
		524 T2D inpatients	90.7% ≥1; 80.0% ≥2	
2007 [27]	1 hospital in 1 middle city	295 diabetes inpatients	89.8% ≥1	Peripheral neuropathy: 69.2%; Hyperlipidemia: 64.1%; Macrovascular: 8.16%; Microvascular: 30.27%; Both macrovascular and microvascular:51.36%
2007–2008 [40]	1 tertiary hospital	489 diabetes inpatients	86.3% ≥1; 46.7% ≥3	
2008–2009 [64]	12 hospitals in 8 cities	1193 T2D outpatients		Peripheral neuropathy:17.0% in the total population;18.3% in the known DM group and 6.4% in the new DM group
2009 [65]	City and village of Ningde Area	5385 adult She ethnic minority		Cardiovascular: 7.4%; Liver dysfunction: 19.4%; Hyperuricemia: 6.2%
2009 [32]	1 southern city	471 diabetic residents		Retinopathy: 24.4%
2005–2010 [37]	1 hospital in 1 middle-income city	2010 general patients over 60 years old		Peripheral arterial disease: 24.1%; Retinopathy: 26.4%; Neuropathy: 20.8%; Macroangiopathy: 34.6%;
2000–2011 [39]	105 hospitals across Guangdong province	3173 T1D patients		Diabetic ketoacidosis: 50.1%; Retinopathy: 8.1%; Nephropathy: 20.7%; Neuropathy: 11.1%
2011–2012 [33]	60 hospitals across Guangdong Province	4616 T2D patients		Diabetic peripheral neuropathy: 31.8%(abdominal obesity), 28.4%(without abdominal obesity) and 10.2% (without obesity)
2011–2012 [34]	62 hospitals across Guangdong Province	2954 T2D outpatients with a BMI ≥25		Stroke: 9.2%; Retinopathy: 20.9%; Nephropathy: 22.1%; Diabetic peripheral neuropathy: 31.6%
2005–2012 [42]	1 southern city	3301 T2D patients		Neuropathy: 19.4%; CKD: 27.1%; Albuminuria: 25.2%
2011–2012 [35]	2 northern city (REACTION study)	18,696 inhabitants over 40 years old (5126 with diagnosis of diabetes)	88.8% ≥ 1, 53.2% ≥ 2	
2011–2013 [41]	8 hospitals from 8 regions	452 diabetic foot ulcer patients	Annual mortality: 14.4%	31.6% new ulcers; Annual incidence of amputation: 5.1%; Nephropathy: 43.1%; Retinopathy: 40.9%; Cataracts: 13.3%
		881 diabetes mellitus patients	Annual mortality: 2.8%	8.1% new ulcers; Annual incidence of ulceration: 8.1%; Nephropathy: 21.7%; Retinopathy: 24.9%; Cataracts: 8.5%

Diabetic retinopathy (DR), diabetic peripheral neuropathy (DPN) and diabetic nephropathy (DN) were reported to be the most common complications. A meta-analysis with 329,316 samples summarized that the prevalences of DR, non-proliferative diabetic retinopathy (NPDR) and proliferative diabetic retinopathy (PDR) were respectively 23.0, 19.1, and 2.8% among diabetic population in China [28], which totaled about 10 million DR patients [29]. The proportion of DR dropped in the past decade in China but was still higher than that in Europe or African [17, 29–31]. By contrast, visual impairment due to DR (8.0%) was relatively uncommon as compared with those in Western countries [5, 29]. The prevalence of DPN was 17.8 and 69.2% among outpatient visits and hospitalizations for diabetes patients respectively in 2007 [24, 26]. In addition, DPN was more prevalent among patients over 60 years old or with BMI over 25 (24.1 and 31.6%) [32, 33]. 10.7% of Type 2 diabetes (T2D) patients had DN in 2007 [24] and proportion of patients with albuminuria was 25.2% [30, 34]. Other complications such as diabetic macrovascular, diabetic microvascular and cardiovascular complications also had high prevalence among Chinese diabetes patients [24].

Diabetic foot ulcer (DFU), which causes great loss to the quality of life, had higher incidence in Chinese diabetes population than those in Western countries. According to a cohort study, 31.6% of patients with diabetic foot ulcer developed new ulcers during follow up period and the annual incidence of amputation is 5.1%, suggesting poor management of DFU. Patients with foot ulcer also had higher prevalence of nephropathy and retinopathy and the annual mortality was five time of that for DB patients [35].

Although International Diabetes Federation reported about 95% diabetes belong to T2D [36], type 1 diabetes (T1D) patients also need attention. About 50.1% of T1D patients in China suffered from diabetic ketoacidosis [27], which was higher than that in US [30]. The prevalences of DR, DN and DPN among T1D patients were 8.1, 20.7, and 11.1% respectively [37].

#### Risk factors for diabetic complications

Male, age and duration of diabetes were important risk factors for most diabetic complications. Generally, male had higher risks for developing diabetic complications, except for DPN [38]. A male diabetic patient was 2.06 times more likely than a woman to develop a foot ulcer [35] while DPN was significantly more prevalent among female patients (59.1% vs 40.9%) [39]. Age was an independent risk factor for most kinds of complications. For example, the peripheral arterial disease (PAD) prevalence was 15.5% among patients aged 60–69 years, comparing to 48.6% among patients older than 80 years [32] and the odds ratio for age was 1.01 for DPN [39, 40]. In contrast, the prevalence of diabetic ketoacidosis among T1D patients decreased with age from 69.2% for patients aged 1–9 years to 21.0% for those older than 60 years old [37]. The risk of developing diabetic complications increased with the duration of diabetes [24, 39, 41]. For patients diagnosed with diabetes for less than 2 years, the age-adjusted prevalence for complications was 30.8%, comparing with 74.1% for patients diagnosed with diabetes for over 15 years. More specifically, the prevalence of cardiovascular disease (CVD) and DR increased from 18.1 to 42.0% and 3.3 to 37.2%, respectively [24].

Geographic variation existed in the prevalence of diabetic complications. The prevalence of DR was higher in the Northern regions of China when compared with Southern regions (1.4% vs. 0.7% among general population and 26.5% vs. 15.7% among diabetic population) [23] and similar trend applied to DPN [40]. Even though the urban population had a higher prevalence of diabetes, patients with diabetes in rural regions had more risk to have complications. The risk for a diabetic patient living in rural area to develop a DFU was 2.23 times that of patient living in city [35] and the prevalence rate of DR was higher among rural diabetic population than that in urban (29.1% vs. 18.1%) [23, 41]. Dietary structure, low diagnosis rate, lack of knowledge for prevention and poor medical care conditions in rural may attribute to the higher prevalence of DBC [41].

Lifestyle also had an influence on diabetic complications and diabetes patients with obesity had higher prevalence of complications. The prevalence of DPN was 31.8, 28.4 and 10.2% among diabetes patients with abdominal obesity, without abdominal obesity and without obesity, respectively [33, 39]. Smoking history gave rise to significant increase of PAD development in T2D patients [32].

Low socioeconomic status (SES) was associated with adverse cardiovascular risk factor patterns and poor outcomes in patients with diabetes. A cross-sectional, multicenter study during 2010–2011 found out that the least educated patients had the highest chances of developing CVD, cerebrovascular diseases, and DR. And lowest household income was associated with highest prevalence of DR and DN [23].

As for other biochemical indicators, insulin level was associated with increased risks for foot ulceration (OR: 3.14) [35]. Elevated blood pressure was significantly associated with DR (OR: 1.64), DN (OR: 3.16), CVD (OR: 2.71), and stroke (OR: 1.90), after adjusting for age, gender, duration of diabetes, and HbA1c [39]. Elevated triacylglycerols was significantly associated with DR (OR: 1.29) and DN (OR: 1.30) [39].

It is also not surprising that some complications were independent risk factors for other complications. One study found that independent risk factors for DPN were DR (OR: 3.10), DN (OR: 2.02), DFU (OR: 3.22), dyslipidemia (OR: 1.67) and history of coronary heart disease (CHD) (OR: 1.43) [37, 39].

#### Awareness and services utilization

The REACTION study reported that the awareness rate of diabetes was 36.3%. same study reported that 27.9% of diabetes patients received treatment and only 34.7% of the patients receiving treatment had their conditions under control [42]. The situation was even worse for diabetic complications. The treatment rate was only 15.3% in patients with DPN in 2011–12 [39]. In addition, only half of the diabetes patients adhered to diet and exercise recommendations. Three-quarters fully complied with prescribed medication, while almost one-fifth of patients said they never followed treatment recommendations from their doctors or nurses [43].

Although diabetes patients in urban regions have better awareness and services utilization than those in rural areas, lack of knowledge for prevention and disease management and delayed treatment are also common in Chinese cities [41]. A research indicated that 43.2% of DR patients had never been examined in urban areas, and among those receiving examination, 66.7% had not been examined as frequently as recommended by clinical guidance. The situation was worse in rural regions that 68.7% hadn’t been examined [19].

### Economic burden associated with DB and DBC

#### Direct economic burden and effects on catastrophic health expenditure and impoverishment

Direct economic burden refers to the expense spent on health care services (direct medical expense) and other direct non-medical expense such as the expense of travel and food when seeking health care services.

The annual direct medical expense per patient increased dramatically with the number of diabetic complications [37, 44]. For T2D patients with complication(s), the annual direct medical expense in 2007 was USD 1895^1^ per patient, compared with USD 510 for those without complications [16], which was consistent with findings from research in Europe [24]. Meanwhile, the annual medical expense for hospital admissions for patients with complications was USD 830, compared with USD 491 for those without complications in 2007 [27]. Direct medical expense varied among different kinds of complications. Patients with microvascular and macrovascular complications had higher annual medical expense for hospitalizations (USD 1041) than those with macrovascular (USD 822) and microvascular disease (USD 735), and those without both (USD 493) in 2007 [27] whereas CVD, DR, and DPN had the highest annual medical expense for outpatient visits [25, 45].

Empirical research on the direct medical expense of DB and DBC is extremely scarce and national study on these issues is absent, despite their major significance on diabetes patients’ welfare. We conducted an analysis using claims data from the Urban Employee Basic Medical Insurance scheme in Hangzhou city, in eastern China in 2011 and the results were reported in Table 2 [46]. Patients with DN had the highest annual medical expense (USD 3466 in 2011^2^), followed by CVD (USD 3074). Annual medical expense was around USD 2450 for patients with DR, DPN and DFU. Medical expense for OP visits distributed similarly across different complications but the medical expense for hospital admissions showed great variation, with CVD admissions associated with an average of USD 3545 per hospitalization stay while DR admissions at only USD 1092. However, these results need to be interpreted with caution since only a relatively small sample had hospitalizations so our estimates can be quite sensitive. On a per patient basis, medical expense for OP visits constitute the main burden. In general, the patients paid approximately 30% of medical bill by out-of-pocket. Again, this result cannot be generalized to the whole nation as the sample are individuals who are insured under the Urban Employee insurance scheme, which is the most generous among the three public insurance schemes in China, the others being the Urban Resident Basic Medical Insurance Scheme and the Rural Health Insurance Scheme.

**Table 2:** Direct medical expenses for patients with different diabetic complications in Hangzhou City, 2011

	DR	DPN	Hypertension	CVD	DN	DFU
# of patients	533	1124	1078	593	242	347
# of patients with hospital admission	24	33	13	35	16	1
Average direct medical expense (USD)						
Per OP visit	61	62	65	65	80	59
Per hospital admission	1092	2448	1764	3545	1858	825
Average annual direct medical expense (USD)						
Overall (per patient)	2455	2451	2299	3074	3466	2459
OP visits (per patient)	2389	2286	2250	2601	3220	2454
Hospital admissions (per patient)	66	165	49	473	246	5
OOP payment rate (%)	30.4	29.5	30.2	30.0	29.9	26.4

As for the composition of direct medical expense for diabetes related care in urban China, existing studies showed that 73–81% had been for treatment of diabetic complications [16, 25], indicating the great economic burden of diabetic complications. Further, existing empirical evidence showed that medications accounted for the biggest share of treatment, followed by tests [26, 46–48].

Diabetes and its complications exposed patients to significant financial risk. Tang et al. reported that annual direct medical expense as a share of average income per capita was 162.2% for patients with diabetic complications, and 32.0% for those without complications [49]. The share was also higher in rural area (163.4%) compared to urban regions (108.65%), largely because income was relatively lower in rural regions [50]. With reimbursement from the public medical insurance schemes, patients’ financial burden was reduced but out-of-pocket payment (as co-payment) for inpatient care remained at 40.4 and 44.6% for patients with and without complications, respectively [27].

Only two research reported the direct non-medical expense for DBC patients in China. An estimation was made by using human capital model and data from the National Health Service Survey that the ratio of direct medical to non-medical expense was 1:0.35 for diabetes care in China, and this indicator was reported to be 1: 0.56 in rural China [48, 51, 52].

#### Indirect burden on patients with DBC

Indirect economic burden refers to the loss in labor productivity due to diseases measured in terms of wages. However, no research has presented such indicator and we used years of life lost due to premature mortality (YLL) instead. The crude number of YLL of diabetes in Jiangsu Province in 2010 was 94.44/100,000, and the age-standardized YLL was 84.10/100,000. More specifically, the YLL was significantly higher for female patients with diabetes and for those residing in the urban areas. The estimation of indirect burden of diabetes in Jiangsu Province was USD 1.1 billion in 2010^3^ [53].

#### Economic burden to health systems

Given the estimation of 11–12 million T2D patients suffering from at least one diabetic complication and about 6 million suffering from more than one complications, the estimated health expenditure of T2D-related complications across mainland China could be a huge economic burden to China’s health system [24]. The total health expenditure for T2D and its complications in 2007 was estimated to be USD 339 billion, accounting for 22.3% of total health expenditure of China and would almost be doubled in 2030 [25].

### Health systems and policies relevant to DB/DBC detection, management and treatment

#### Public health services

The number of health policies and strategies on diabetes and its complications at the national level was very limited. In 2015, the State Council released the Guidance on the Implementation of the Coordinated Care and Referral System and identified diabetes and hypertension as the two prioritized diseases for piloting [54]. It aimed to rationalize service utilization by encouraging patients with common and chronic diseases, such as diabetes and hypertension, to use primary health care facilities, which acted as gatekeepers for referral to secondary/tertiary care when needed. Management for such conditions was also the major responsibility of primary care providers. This policy aimed to enroll at least 40% of diabetes and hypertension patients under the management of primary healthcare institutions by the end of 2017. Patients were expected to have electronic records (which can be shared in the whole health service systems), and receive routine treatment, regular follow-up, physical examinations, and health educations on diet, etc. Early screening for complications can also be introduced at primary or secondary healthcare institutions [55].

#### Health insurance

Over 98% of the population in China has now been covered by one of the three public Basic Medical Insurance Schemes—Urban Employee, Urban Resident and Rural Resident, with the latter two largely financed by general tax revenue [21]. In order to provide further financial protection to patients with catastrophic health expenditure, the Chinese government introduced the Insurance Program for Catastrophic Diseases (IPCD) in 2012 to cover medical expense associated with a list of health conditions that incur catastrophic health expenditure. Unfortunately, despite the heavy burden of diabetes and its complications, they were not covered by this program at the national level. However, several inspiring policies have been implemented in some provinces.

Guangzhou, with a GDP of USD 22,217^4^ per capita in 2015 and the provincial capital of Guangdong Province located in Southern China, announced an extra pooling of fund as an extension of the Urban Employee’s Basic Medical Insurance (UEBMI) to provide further reimbursement for outpatient medical expense for patients with chronic disease(s). After getting diagnosed and registered with designated hospitals, patients having diabetes, hypertension, Alzheimer’s disease, liver cirrhosis or other kinds of chronic diseases (in total 20 eligible chronic diseases) were entitled to get an 85% or 65% reimbursement rate at primary healthcare institutions or other levels of hospitals respectively on their medical expense. Yet medical expense for treating diabetic complications were not covered by this policy [56].

Tianjin, located in Northern China with a GDP of USD 17,165 per capita in 2015, has conducted a pilot in one secondary hospital from 2014. This pilot has innovatively used reimbursement policy on patients and payment reform on provider side to incentivize better management for diabetes. More specifically, after getting diagnosis of diabetes in designated hospitals and approval from the insurance agency, patients can register in the pilot hospital and enjoy a higher reimbursement rate for outpatient services of diabetes in this hospital. The pilot hospital is paid by capitation (USD 1613 per patient per year) for the registered patients’ outpatient services for diabetes (treatment for diabetic complications is excluded) [57]. In order to attract more patients, the pilot hospital adopted a series of innovations in service delivery. For instance, patients can make appointment for their outpatient visits online, experts were invited from tertiary hospitals to provide consultation for patients on-site, etc. Moreover, the pilot hospital laid more attention on the follow-up and education on patients so as to achieve better outcome and satisfaction. An internal report found that more than 95% of the patients with diabetes were more satisfied for the services provided in pilot hospital and the control rate of patients’ blood glucose was improved after the pilot. In 2015, the pilot was extended to 6 more hospitals.

Xiamen, the provincial capital of Fujian Province located in Southern China with a GDP of USD 14,511 per capita in 2015, was one of the pilot cities of the Coordinated Care and Referral System. Reimbursement from the basic medical insurance became the key to the implementation of this system in that patients received higher reimbursement rate if they seek care at primary healthcare institutions, as a way to incentivize patients to use primary care. Further, registration fees would be waived if the patient were referred from primary healthcare institution to higher levels of healthcare institution. Additionally, medicines prescribed during outpatient visit at primary healthcare institutions for hypertension or diabetes can get full reimbursement from insurance. It is worth noting that 46 generic medicines were eligible for patients with diabetes, among which Traditional Chinese Medicines, as well as medicines to prevent renal disease were included (Renal disease is one of the common complications of diabetes). However, insulin hasn’t been included in this reimbursable medicine list [58, 59].

Ningxia, a province located in the North-West of China and has a GDP of USD 6716 per capita in 2014, introduced a risk pooling fund as part of their Urban Employee and Urban/Rural Residents Basic Medical Insurance schemes (note: Ningxia has integrated the three public insurance schemes to two, one for urban employees and the other for the rest) to cover outpatient services of catastrophic diseases. Twenty-eight major diseases, such as cancer, organ transplant, dialysis, coronary heart disease, diabetes and its complications, hypertension and its complications, were identified for inclusion for coverage. After an USD 81 deductible, patients can get 50–75% of their outpatient expense reimbursed. Since the financing mechanisms were different between the two schemes, the maximum reimbursement amount differed. For diabetes, the annual cap was set at USD 161 and USD 113 in 2014 for the Urban Employee and Urban/Rural Resident Basic Medical Insurance schemes, respectively. For patients with diabetic complication, the annual caps were USD 484 and USD 339 accordingly. Patients need to apply for this benefit package, and when approved, will be designated a primary and a secondary providers for treatment [60].

## Discussions

### Primary and secondary prevention

Healthy lifestyle reduces the risk of diabetes, it is also one of the protective factors for diabetes and its complications [61, 62]. For instance, each 5 kg/m^2^ decrease in BMI will prevent about 30% overall mortality for diabetic population [34]. Other factors such as early diagnosis of diabetes, early treatment with compliance and early screening of complications on high risk patients with diabetes also delay the deterioration of diabetes and its complications.

However, the lack of knowledge about diabetes and its complications among Chinese population brought great challenges to the primary and secondary prevention of diabetes and its complications. Without knowledge of diabetes and its prevention and treatment, 64 to 76% of diabetes in China was undiagnosed which left the “patients” without any treatment. And nearly 80% of patients with diabetes had poor glucose control [19], and adherence to diet, exercise or treatment recommendations from doctors or nurses was far from satisfactory [43].

Education about the basic knowledge of diabetes, nutrition, healthy lifestyle and prevention of diabetes should be provided regularly through multiple media such as lectures, TV news, radio, internet, etc. The Shanghai government distributed pots for controlling the amount of oil dispenses, rulers for measuring waist, and salt-control spoons to all residents for free. It is a good example of intervention to advocate and practice healthy diet. Furthermore, lectures and follow up visits/phone calls from hospitals should be provided to patients with diabetes and their families with the help of electronic patient record. This experience is also applicable to other developing countries where the lifestyle is changing rapidly with the globalization trend but the people’s awareness of disease hasn’t been synchronized.

Another challenge to the prevention of diabetes and its complications is the missing of an early screening mechanism on high risk population and a patient management program. The Asia Pacific Type 2 Diabetes Policy Group (APDPG) recommended that diabetes complications should be assessed at an interval of 1–2 years and a number of tests or examinations have been proved to be cost-effectiveness on early detecting diabetic complications [32, 43]. And patient management programs, including regularly follow up on the compliance to treatment, also contribute to the better outcome of patients. The biggest problem was no government department or clinical unit is taking the responsibility to organize screening or patient management for diabetes populations. And lack of funding was also a barrier to support the regular screen and patient management in long term.

By introducing a combination of performance assessment system and provider payment method aiming at clinical outcome, the pilot in Tianjin showed the possibility and benefits of establishing a screening and patient management system by healthcare providers. Countries with uneven development between treatment and prevention, should also take actions to advocate the early screening and patient management by strengthening its public health sector or providing incentives on outcome-oriented health system.

### Health systems development and strengthening

With an increasingly burden of non-communicable diseases such as diabetes and its complications, an integrated services delivery system centered on primary level is recommended to promote education, early case-detection and screening, patient management, referral and care-coordination between primary, secondary and tertiary health care providers.

The challenges for developing integrated care for diabetes and its complications are numerous in China. First, China’s health delivery system put too much emphasis on hospitals while limited resource or attention was allocated to primary care providers or public health sector. This long-lasting but uneven developed delivery system will take a number of years to reverse [63]. Second, health human resource at the primary care providers is inadequate in both numbers and quality. Third, since the majority of providers are still paid by fee-for-service payment methods, it creates strong disincentives for providers of different levels to coordinate or integrate care. Likewise, providers are not incentivized to deliver case management or prevention. Fourth, except for some isolated cases, the major of medical insurance schemes provide more generous coverage for hospitalizations than OP visits and thus do not provide incentives for patients to seek care at the primary care levels or to engage in prevention and health promotion. All these are hypothesized to have contributed to the poor management of diabetes and the growth in diabetic complications and poor management.

However, opportunities exist in addressing these challenges. For example, as described, a number of locations have realized the high financial risk and potential health outcome loss faced by patients with diabetes and complications and introduced financing and reimbursement policies to address these dire outcomes. The central government should conduct careful evaluations of these local interventions, draw lessons on what works and what does not work and provide guidance for the rest of the countries to learn from.

Provider payment methods also need to be reformed in order to promote integrated delivery. In particular, fee-for-service needs to be changed to population-based capitation payment methods, which pays an integrated delivery system a fixed amount for caring (from prevention to treatment across all levels of facilities in the integrated delivery system) a patient for a fixed period of time, and hold these providers accountable for their care using a combination of performance-based payment, audits, etc. China has already announced its policy to reform provider payment and thus the window of opportunity has been created.

To enhance integrated delivery targeting priority chronic diseases, such as diabetes and its complications, human resources are critical. China needs to train more general practitioners with proper qualifications and be able to retain them. Over time, their functions will be more comprehensive so that some of the current care/treatment delivered in secondary care can be shifted to them, and they will be the case-manager of integrated care for the patients. Again, China has embarked on significant training for GPs.

Finally, a functioning management information system with comprehensive registry of all patients with diabetes and those with complications is required. China has invested a lot in management information system with electronic records created for patients with diabetes. However, this system needs to be able to flow across the different levels of providers and unique to an individuals in order to facilitate integrated care.

## Conclusions

In sum, China faces significant disease and economic burdens due to diabetes and its complications. Significant gaps exist in prevention, management and treatment and providing patients with financial risk protection. These gaps need to be closed through a number of systemic level interventions. Efforts should be invested in education, early screening mechanism and patient management programs to improve the primary and secondary prevention of diabetes and its complications. An integrated services delivery system centered on primary level is recommended to promote education, early case-detection and screening, patient management, referral and care-coordination between primary, secondary and tertiary health care providers.

## Additional file

Additional file 1: Searching Strategy. Searching strategies used for night online databases (including two in Chinese) have been listed. (DOCX 18 kb)

## Abbreviations

CVD: Cardiovascular disease
DB: Diabetes
DBC: Diabetic complications
DFU: Diabetic foot ulcer
DN: Diabetic nephropathy
DPN: Diabetic peripheral neuropathy
DR: Diabetic retinopathy
NPDR: Non-proliferative diabetic retinopathy
PAD: Peripheral arterial disease
PDR: Proliferative diabetic retinopathy
SES: Socioeconomic status
T1D: Type 1 diabetes
T2D: Type 2 diabetes

## Footnotes

1 USD1 = CNY7.3 (2007)

2 USD1=CNY6.5 (2011)

3 USD1 = CNY6.8 (2010)

4 USD1 = CNY6.2 (2015)
