# Supplementary material for: Complications of diabetes in China: health system and economic implications
Source: BMC Public Health. 2019 Mar 6;19:269. doi: 10.1186/s12889-019-6569-8 (PMC6414024; doi:10.1186/s12889-019-6569-8)
Supplement: Supplementary file 1 — Searching Strategy. Searching strategies used for night online databases (including two in Chinese) have been listed. (DOCX 18 kb) [file 12889_2019_6569_MOESM1_ESM.docx]

Additional file 1 Searching Strategy

1. PubMed : (((China[Title]) AND (“Diabetes Complication*”[Title/Abstract] OR “Diabetes Related Complication*”[Title/Abstract] OR “Complication* of Diabetes Mellitus”[Title/Abstract] OR “Diabetes Mellitus Complication”[Title/Abstract] OR “Diabetic Angiopathies”[Title/Abstract] OR “Diabetic foot”[Title/Abstract] OR “Diabetic Retinopathy”[Title/Abstract] OR “Diabetic Cardiomyopathies”[Title/Abstract] OR “Diabetic Coma”[Title/Abstract] OR “Hyperglycemic Hyperosmolar Nonketotic Coma”[Title/Abstract] OR “Diabetic Ketoacidosis”[Title/Abstract] OR “Diabetic Nephropathies”[Title/Abstract] OR “Diabetic Neuropathies”[Title/Abstract] OR “Fetal Macrosomia”[Title/Abstract])) AND (“Secondary prevention” OR “Screening” OR “Community management” OR “Diagnosis” OR “treatment”)) AND (“Prevalence” OR “Cost” OR “Economic burden” OR “Economic evaluation” OR “Health insurance” OR “medical insurance”)
2. Cochrane library: China in Record Title and "Diabetes Complication*" OR "Diabetes Related Complication*" OR "Complication* of Diabetes Mellitus" OR "Diabetes Mellitus Complication" OR "Diabetic Angiopathies" OR "Diabetic foot" OR "Diabetic Retinopathy" OR "Diabetic Cardiomyopathies" OR "Diabetic Coma" OR "Hyperglycemic Hyperosmolar Nonketotic Coma" OR "Diabetic Ketoacidosis" OR "Diabetic Nephropathies" OR "Diabetic Neuropathies" OR "Fetal Macrosomia" in Title, Abstract, Keywords and "Secondary prevention" OR "Screening" OR "Community management" OR "Diagnosis" OR "treatment" and "Prevalence" OR "Cost" OR "Economic burden" OR "Economic evaluation" OR "Health insurance" OR "medical insurance" in Trials'
3. EMBASE(OVID): China.ti. and (Diabetes Complication* or Diabetes Related Complication* or Complication* of Diabetes Mellitus or Diabetes Mellitus Complication or Diabetic Angiopathies or Diabetic foot or Diabetic Retinopathy or Diabetic Cardiomyopathies or Diabetic Coma or Hyperglycemic Hyperosmolar Nonketotic Coma or Diabetic Ketoacidosis or Diabetic Nephropathies or Diabetic Neuropathies or Fetal Macrosomia).ab. and (Secondary prevention or Screening or Community management or Diagnosis or treatment).af. and (Prevalence or Cost or Economic burden or Economic evaluation or Health insurance or medical insurance).af.
4. Elsevier ScienceDirect: (TITLE(China) and TITLE-ABSTR-KEY(Diabetes Complication OR Diabetes Related Complication OR Complication of Diabetes Mellitus OR Diabetes Mellitus Complication)
5. EBSCO(MEDLINE): TI China AND AB ( “Diabetes Complication*” OR “Diabetes Related Complication*” OR “Complication* of Diabetes Mellitus” OR “Diabetes Mellitus Complication” OR “Diabetic Angiopathies” OR “Diabetic foot” OR “Diabetic Retinopathy” OR “Diabetic Cardiomyopathies” OR “Diabetic Coma” OR “Hyperglycemic Hyperosmolar Nonketotic Coma” OR “Diabetic Ketoacidosis” OR “Diabetic Nephropathies” OR “Diabetic Neuropathies” OR “Fetal Macrosomia” ) AND TX ( “Secondary prevention” OR “Screening” OR “Community management” OR “Diagnosis” OR “treatment” ) AND TX (“Prevalence” OR “Cost” OR “Economic burden” OR “Economic evaluation” OR “Health insurance” OR “medical insurance” )
6. Web of science: TI=(China) AND TS=(“Diabetes Complication*” OR “Diabetes Related Complication*” OR “Complication* of Diabetes Mellitus” OR “Diabetes Mellitus Complication” OR “Diabetic Angiopathies” OR “Diabetic foot” OR “Diabetic Retinopathy” OR “Diabetic Cardiomyopathies” OR “Diabetic Coma” OR “Hyperglycemic Hyperosmolar Nonketotic Coma” OR “Diabetic Ketoacidosis” OR “Diabetic Nephropathies” OR “Diabetic Neuropathies” OR “Fetal Macrosomia”) AND TS=(“Secondary prevention” OR “Screening” OR “Community management” OR “Diagnosis” OR “treatment”) AND TS=(“Prevalence” OR “Cost” OR “Economic burden” OR “Economic evaluation” OR “Health insurance” OR “medical insurance”)
7. WILEY: China in Article Titles AND “Diabetes Complication*” OR “Diabetes Related Complication*” OR “Complication* of Diabetes Mellitus” OR “Diabetes Mellitus Complication” OR “Diabetic Angiopathies” OR “Diabetic foot” OR “Diabetic Retinopathy” OR “Diabetic Cardiomyopathies” OR “Diabetic Coma” OR “Hyperglycemic Hyperosmolar Nonketotic Coma” OR “Diabetic Ketoacidosis” OR “Diabetic Nephropathies” OR “Diabetic Neuropathies” OR “Fetal Macrosomia” in Abstract AND “Secondary prevention” OR “Screening” OR “Community management” OR “Diagnosis” OR “treatment” in All Fields AND “Prevalence” OR “Cost” OR “Economic burden” OR “Economic evaluation” OR “Health insurance” OR “medical insurance” in All Fields
8. WanFang [in Chinese]: Search 1: 题名或关键词:(糖尿病)*关键词:(糖尿病并发症+糖尿病慢性并发症+糖尿病足+糖尿病视网膜病变+糖尿病心肌病+糖尿病昏迷+高渗性非酮症糖尿病昏迷+糖尿病酮症酸中毒+糖尿病肾病+糖尿病神经病变+糖尿病血管+巨大儿)*摘要:(二级预防+筛查+筛检+社区管理+诊断+治疗)*关键词:(患病+成本+费用+经济负担+疾病负担+经济学评价+医疗保险+城镇职工+城镇居民+新农合+城乡居民) * Date:-2016

Search 2: 题名或关键词:(糖尿病)*关键词:(糖尿病并发症+糖尿病慢性并发症+糖尿病足+糖尿病视网膜病变+糖尿病心肌病+糖尿病昏迷+高渗性非酮症糖尿病昏迷+糖尿病酮症酸中毒+糖尿病肾病+糖尿病神经病变+糖尿病血管+巨大儿)*关键词:(二级预防+筛查+筛检+社区管理+诊断+治疗)*摘要:(患病+成本+费用+经济负担+疾病负担+经济学评价+医疗保险+城镇职工+城镇居民+新农合+城乡居民) * Date:-2016

1. CNKI[in Chinese]:Search 1: TI='糖尿病' and KY=('糖尿病并发症'+'糖尿病慢性并发症'+'糖尿病足'+'糖尿病视网膜病变'+'糖尿病心肌病'+'糖尿病昏迷'+'高渗性非酮症糖尿病昏迷'+'糖尿病酮症酸中毒'+'糖尿病肾病'+'糖尿病神经病变'+'糖尿病血管'+'巨大儿') and AB=('二级预防'+'筛查'+'筛检'+'社区管理'+'诊断'+'治疗') and KY=('患病'+'成本'+'费用'+'经济负担'+'疾病负担'+'经济学评价'+'医疗保险'+'城镇职工'+'城镇居民'+'新农合'+'城乡居民')

Search 2: TI='糖尿病' and KY=('糖尿病并发症'+'糖尿病慢性并发症'+'糖尿病足'+'糖尿病视网膜病变'+'糖尿病心肌病'+'糖尿病昏迷'+'高渗性非酮症糖尿病昏迷'+'糖尿病酮症酸中毒'+'糖尿病肾病'+'糖尿病神经病变'+'糖尿病血管'+'巨大儿') and KY=('二级预防'+'筛查'+'筛检'+'社区管理'+'诊断'+'治疗') and AB=('患病'+'成本'+'费用'+'经济负担'+'疾病负担'+'经济学评价'+'医疗保险'+'城镇职工'+'城镇居民'+'新农合'+'城乡居民')
